# Pain Self-Management Behaviors in Breast Cancer Survivors Six Months Post-Primary Treatment: A Mixed-Methods, Descriptive Study

**DOI:** 10.3390/cancers17071087

**Published:** 2025-03-24

**Authors:** Kaitlin M. McGarragle, Sunny Zheng, Lucia Gagliese, Doris Howell, Elizabeth Edwards, Cheryl Pritlove, David McCready, Christine Elser, Jennifer M. Jones, Lynn R. Gauthier

**Affiliations:** 1 Department of Supportive Care, Princess Margaret Cancer Center, University Health Network, Toronto, ON M5G 2M9, Canada; kmcgarragle@live.ca (K.M.M.); ss.zheng@mail.utoronto.ca (S.Z.); doris.howell@uhn.ca (D.H.); beth.edwards@uhn.ca (E.E.); cheryl.pritlove@unityhealth.to (C.P.); 2 Institute of Medical Science, University of Toronto, Toronto, ON M5S 3H2, Canada; 3 School of Kinesiology and Health Science, York University, Toronto, ON M3J 1P3, Canada; gagliese@yorku.ca; 4 Department of Anesthesia and Pain Management, University Health Network, Toronto, ON M5G 2C4, Canada; 5 Department of Anesthesia, University of Toronto, Toronto, ON M5T 2S8, Canada; 6 Department of Psychiatry, University of Toronto, Toronto, ON M5T 1R8, Canada; 7 Department of Anesthesia and Pain Management, Mount Sinai Hospital, Sinai Health System, Toronto, ON M5G 1X5, Canada; 8 Li Ka Shing Knowledge Institute, St. Michael’s Hospital, Toronto, ON M5B 1W8, Canada; 9 Division of Social and Behavioral Science, Dalla Lana School of Public Health, University of Toronto, Toronto, ON M5T 3M7, Canada; 10 Department of Surgical Oncology, Princess Margaret Cancer Center, University Health Network, Toronto, ON M5G 2M9, Canada; david.mccready@uhn.ca; 11 Department of Surgery, Faculty of Medicine, University of Toronto, Toronto, ON M5T 1P5, Canada; 12 Department of Medical Oncology and Hematology, Princess Margaret Cancer Center, University Health Network, Toronto, ON M5G 2M9, Canada; christine.elser@uhn.ca; 13 Division of Medical Oncology, Faculty of Medicine, University of Toronto, Toronto, ON M5S 3H2, Canada; 14 Marvelle Koffler Breast Center, Mount Sinai Hospital, Sinai Health System, Toronto, ON M5G 1X5, Canada; 15 Department of Family and Emergency Medicine, Faculty of Medicine, Laval University, Quebec, QC G1V 0A6, Canada; lynn.gauthier@crchudequebec.ulaval.ca; 16 Michel-Sarrazin Research Team in Psychosocial Oncology and Palliative Care, CHU de Québec-Laval University Research Center, Oncology Division, Quebec, QC G1J 1Z4, Canada; 17 Cancer Research Center, Laval University, Quebec, QC G1R 3S3, Canada

**Keywords:** breast cancer, pain, survivorship, self-management

## Abstract

**Background/Objectives:** One-third of breast cancer (BC) survivors experience chronic treatment-related pain (CTP) that requires multimodal management strategies, which may include pain self-management behaviors (PSMBs). Most studies exploring PSMBs focus on patients with advanced cancer, who may differ from survivors in their pain management needs and access to resources. This mixed-methods study explored PSMBs of survivors of BC, referral sources, and goals for pain relief, and examined the relationship between PSMB engagement and pain intensity/interference. **Methods:** Survivors of BC who were six months post-treatment completed measures assessing their pain intensity/interference and PSMB engagement. Purposive sampling identified a subset of participants who completed interviews, which were analyzed using thematic analysis. **Results:** Participants (*n* = 60) were 60 ± 10 years old. Worst Pain Intensity and Pain Interference were 3.93 ± 2.36 and 2.09 ± 2.11, respectively. Participants engaged in 7 ± 3.5 PSMBs. The most common were walking (76%) and distraction (76%). PSMBs described in the interviews (*n* = 10) were arm stretching and strengthening exercises, seeking specialized pain management services, and avoidance. Most PSMBs were self-directed or suggested by friends. All pain relief goals were to minimize pain interference. PSMB engagement was not associated with Worst, Least, or Average Pain Intensity (all r_s_ ≤ −0.2, *p* ≥ 0.05) but was associated with Pain Interference (r_s_ = 0.3, *p* ≤ 0.01). **Conclusions:** The survivors of BC engaged in many PSMBs, with varying levels of effectiveness and a varying quality of supporting evidence. Most PSMBs were self-directed and some required intervention from healthcare providers or other people, while others required access to limited specialized pain management services.

## Simple Summary

One-third of breast cancer survivors report persistent pain following cancer treatments like surgery, radiation, and chemotherapy. Little is known about what they do to self-manage their pain. Therefore, this study asked breast cancer survivors about the things they do to self-manage their pain, how they were referred to specific pain management strategies, and their goals for pain relief. This study also examined the relationship between engagement in pain self-management strategies and pain intensity and interference. Commonly reported pain self-management behaviors included walking and general exercise, and distraction. Pain interference, but not pain intensity, was associated with pain self-management behaviors, and interviews provided additional context about pain interference reduction as an important pain relief goal, highlighting interference as a clinically important outcome.

## 1. Introduction

Breast cancer (BC) is the most commonly diagnosed cancer in women worldwide [1]. As BC treatment becomes increasingly sophisticated and the number of survivors of BC continues to rise [2], the treatment of persistent and late effects of BC will become increasingly urgent. Chronic treatment-related pain (i.e., pain that persists >3 months post-treatment) is one of the most common long-term effects of cancer treatment [3] and its prevalence among cancer survivors is almost double that of pain in the general population [4,5,6,7]. In survivors of BC, estimates of its prevalence vary from 25% to 75% [8,9,10,11,12,13]. A recent meta-analysis of chronic treatment-related pain following breast cancer surgery reported a median prevalence of 37.5% across 23 studies [14]. Chronic treatment-related pain may be nociceptive, neuropathic, or nociplastic [15]. It commonly occurs in the breast and underarm area, but may also be experienced in other body regions, such as the upper and lower extremities, or throughout the body as widespread muscle and joint pain. It can be influenced by a number of interacting biopsychosocial factors including the disease, treatments (surgery, radiation, chemotherapy, and/or hormone therapy), psychological wellbeing, social support, and socioeconomic factors [16]. It has been shown to persist for months and years following treatment [10,17,18,19,20], can significantly impact patients’ quality of life, has been associated with poorer physical and psychological outcomes in survivors of BC [13,21,22,23,24], and can result in higher healthcare costs [3].

Similar to other chronic conditions, chronic treatment-related pain requires active involvement and self-management by the patient on a daily basis [25]. While there are several working definitions of pain self-management [26,27], self-management in general refers to the medical, life role, and emotional management tasks involved in coping with a chronic condition [28]. For example, self-management among patients with chronic pain may include taking medication to improve pain relief (medical management), pacing energy expenditure to maximize activity engagement (life role management), and eliciting social support to foster positive affect (emotional management). For example, the Symptom Management Theory [29,30] is a comprehensive framework that can be used to understand pain self-management behaviors (PSMBs). According to this framework, symptom management strategies involve assessing a symptom, identifying a goal for intervention, and taking action to achieve a desired outcome [29]. Desired outcomes can include a reduction in symptom intensity, symptom frequency, interference in daily activities, and/or symptom-related distress [30]. As such, PSMBs can be viewed as activities aimed at minimizing pain intensity and/or pain-related impact or interference.

There has been very little research describing the PSMBs that survivors of BC engage in to manage chronic treatment-related pain, and there are no studies to our knowledge that have explored patient-reported PSMBs. Research on PSMBs in people with cancer has focused on healthcare provider (HCP)-designed and led specific PSMB interventions [29,30,31,32]. For example, many PSMB interventions use HCP-coaching and informational tools such as booklets to educate patients about specific PSMBs such as taking pain medication [33]. However, this approach may neglect the patient perspective and risks overlooking PSMBs that people engage in that may not necessarily be known to HCPs. Existing knowledge about PSMBs in people with cancer is also derived largely from studies of people with advanced disease or people within the first 3 months after surgery [31,32,33,34,35]. These populations may anticipate and evaluate pain differently than disease-free cancer survivors. In addition, the meaning that cancer survivors attribute to pain may be an important factor in determining their pain-related help-seeking and avoidance behaviors [36] and may differ from that of patients at other phases of the cancer trajectory. Cancer survivors may also lack access to specialized pain management resources post-primary treatment and, therefore, may seek and engage in different PSMBs compared to people with advanced cancer who may have more frequent access to specialized pain management at cancer centers. Finally, most studies examining PSMBs have only included patients with moderate to severe pain [31,32], thereby excluding patients who may be effectively self-managing pain and who may be key informants about the types of PSMBs that cancer survivors engage in.

Of additional importance are addressing goals for pain relief and PSMB referral sources. The goals for pain relief among people with active cancer may reflect a desired reduction in pain intensity to allow them to participate in basic activities of daily living or other valued activities [37,38]. While the goals for pain relief from PSMBs among cancer survivors may be similar, no studies have reported on the relationships between patient-reported or -initiated PSMBs and pain intensity and interference outside of a structured intervention context (e.g., a patient education intervention). Patient-reported goals for pain relief from PSMBs among cancer survivors have yet to be investigated. More broadly, patients with cancer may seek information about complementary and alternative medicine (CAM) for symptom relief from their HCP, the internet, or word-of-mouth, particularly that of close family members or friends [39,40,41,42]. Understanding referral sources for PSMBs may provide valuable insight into why cancer survivors engage in some PSMBs and not others. Identifying PSMBs that survivors of BC perceive to work well and those that do not will help to advance our understanding of the range of behaviors in which survivors engage and advance efforts to measure it. It could also contribute to the development of pain self-management interventions to improve the quality of life of survivors of BC and lower morbidity post-treatment. Therefore, the objectives of this study were to: (1) describe the PSMBs in which survivors of BC engage; (2) to determine the relationship between PSMBs and pain intensity and interference; (3) to describe the goals for pain relief of survivors of BC; and (4) to describe PSMB referral sources.

## 2. Materials and Methods

This study used a qualitative descriptive sequential explanatory mixed-methods design [43,44]. Participants first completed questionnaires which were then used to purposively sample a subset of participants, who then completed individual semi-structured, qualitative descriptive interviews [45].

This study was approved by the Research Ethics Board of the University Health Network (REB #: 13-7178). The sample for the present study was drawn from a larger longitudinal study of pain in the first year following BC treatment. As part of this larger study, participants were recruited from Princess Margaret Cancer Center in Toronto, Canada between July 2014 and July 2016. Patients eligible for this study were female patients with BC who were ≥18 years old; 6 months (±2 months) post-primary treatment for BC (i.e., post-radiation and/or chemotherapy); and able to provide informed consent and complete study questionnaires in English. Patients with metastatic disease or documented cognitive impairment identified by a HCP or medical chart were not approached. Patients who consented were administered a cognitive screen, the Short Orientation-Memory-Concentration test (SOMC) [46], by a research assistant (RA), and those who scored <20 were deemed ineligible. The SOMC is a validated 6-item measure aimed at detecting cognitive impairment [46] and has been used with patients with cancer [47,48,49]. At the time of follow-up (i.e., 6 months post-primary radiation and/or chemotherapy) and prior to receiving their study questionnaire in the mail, participants were contacted by phone to update relevant demographic and clinical information such as any medications they were taking.

Participants were eligible for the qualitative phase of the study if they had agreed to future contact for an interview at baseline and reported engagement in one or more PSMBs at the time of follow-up. Purposive sampling was used to categorize participants according to high (≥4/10) and low (≤3/10) pain intensity [12,33], as measured using the Worst Pain in the last 24 h item from the Brief Pain Inventory (BPI) [50] and high and low engagement in PSMB, as measured using the Pain Self-Care Behaviors Questionnaire (PSCBQ) (Miaskowski, written communication, 2014), after they returned their questionnaires. As no standardized scoring criteria for the PSCBQ exists, a mean split was used (high: above the overall sample mean for the PSCBQ item ‘Total Number of PSMB’; low: below the overall sample mean for ‘Total Number of PSMB’). This was done in order to capture participants who were engaging in many PSMBs and those who were engaging in very few. Care was taken to ensure representation across all purposive sampling categories. Based on this sampling method, those who were identified as eligible were contacted by the study RA, who explained the objectives of the interview. Informed consent was obtained from participants who agreed.

The interview guide was informed by the SMT [28,29], see Appendix A. Qualitative rigor was established in several ways. Credibility was established through peer debriefing among the research team. Regular meetings were held to discuss any challenges that arose during data collection. Dependability was established through external audit, and researchers (BE, CP) with qualitative methodology expertise who reviewed the interview guide and the first two transcripts provided feedback about question probes and overall interviewing style. Internal validity was established through independent coding by two authors and consultation with a third author regarding discrepancies in coding (see Section 2.4).

### 2.1. Measures

#### 2.1.1. Demographic and Clinical Information

Demographic information collected from participants included age, ethnicity, education, marital status, employment status, and living arrangement. Disease and treatment-related information included type of surgery, adjuvant treatment, and current pain- and non-pain-related medications.

#### 2.1.2. Engagement in Pain Self-Management Behaviors

The Pain Self-Care Behaviors Questionnaire (PSCBQ) (Miaskowski, written communication, 2014) is a 25-item measure that identifies the PSMBs in which participants engage, as well as the effectiveness of each PSMB for pain relief. It includes items such as taking pain tranquilizers (medical management), reducing work hours (life role management), and doing relaxation exercises or meditation (emotional management). Participants indicate whether or not they have engaged in a given PSMB (yes/no) in the past week. For endorsed items, participants are asked to rate the effectiveness of that PSMB in helping their pain on a scale from 0 to 10. The PSCBQ is the only measure that comprehensively assesses PSMBs. It has been used with patients with cancer [51] and in patients with end-stage liver disease [52].

Three scores were calculated from the PSCBQ responses. The first two were based on scoring used by Hansen et al. (2014) [52]: Total Number of PSCBQ-PSMBs (sum of all endorsed items) and Mean Effectiveness (average effectiveness of all endorsed items). In addition, a third score, Maximum Effectiveness (highest reported effectiveness score per participant who endorsed ≥1 item) was calculated in order to ensure that participants who were engaging in fewer PSMBs but finding them effective were represented. Cronbach’s alpha for Total Number of PSMBs was 0.769. There were insufficient cases to report Cronbach’s alpha for Mean or Maximum Effectiveness.

#### 2.1.3. Pain Measures

The Brief Pain Inventory (BPI) is a validated measure of cancer pain intensity and interference [50]. Participants rate their worst, least, average, and current pain intensity, as well as interference from pain in seven physical and psychological domains, on numeric rating scales ranging from 0 to 10, with higher scores reflecting worse pain intensity and interference. The BPI has been extensively validated in patients with cancer [53,54,55]. Cronbach’s alphas for the BPI Intensity and Interference subscales in the present study were 0.960 and 0.940, respectively.

### 2.2. Semi-Structured Interview Schedule

Semi-structured interviews were conducted by one author (KMM) and were audio recorded and transcribed verbatim, excluding patient-identifying information. Interviews took place over the phone and in-person when possible and lasted approximately 40 min. The interviewer was experienced in qualitative methodology and was familiar with topics in cancer survivorship and pain research.

The interview guide was developed and refined by KM and LRG using the SMT [28,29] and reviewed by CP and EE who have expertise in qualitative research in people with cancer. Interviews focused on the following: (1) what behaviors participants engaged in to manage their pain; (2) goals for pain relief; and (3) referral sources for PSMBs. Examples of interview questions include ‘Do you use any strategies to help manage your pain? If so, what do these include?’, ‘What are your goals for pain relief?’, and ‘How did you come to know about these strategies?’.

### 2.3. Quantitative Data Analysis

Data were checked for normality using the Shapiro-Wilk test and data entry errors, and descriptive statistics were used to describe participants’ demographic and clinical characteristics, pain, and engagement in PSCBQ-PSMBs. Independent sample *t*-tests and chi square analysis were used to assess differences between participants who returned questionnaires and those who did not. *T*-tests were used to assess continuous variables and chi square analysis was used to assess categorical variables. Spearman’s rank order correlations were computed to determine the relationship between engagement in PSCBQ-PSMBs and BPI Pain Intensity and Interference. Independent samples *t*-tests were also used to examine differences in pain (using the BPI Worst Pain item) between participants who engaged versus those who did not engage in PSCBQ-PSMBs. Data analysis was conducted using SPSS Version 20.0 for Windows.

### 2.4. Qualitative Data Analyses

Qualitative data were analyzed using thematic analysis described by Braun & Clarke (2006) [56]. Interviews were independently coded by two authors (KM, SZ) and discussed to establish internal validity. When disagreement between the two coders (KM, SZ) occurred, a third author (LRG) was consulted until agreement was reached. Interviews were first coded deductively using a preliminary codebook that included known PSMBs from the existing literature [32,33,57,58]. Interviews were then coded inductively, and new categories and codes were added to the existing codebook as they emerged. Once a final version of the codebook that included a priori and new themes was established, a second round of coding was completed. Interviews were conducted until saturation was reached, meaning no new themes (i.e., no new categories of PSMB) were identified [59].

## 3. Results

### 3.1. Quantitative Outcomes

#### 3.1.1. Demographic and Clinical Characteristics

Participants’ demographic and clinical factors for the current study sample (*n* = 60) are presented in Table 1. The mean age was 60 ± 9.88 years old. The majority of participants were White and highly educated (college degree or higher). Most underwent radiation and/or chemotherapy and reported taking hormone therapy at the time of follow-up.

**Figure 1:**
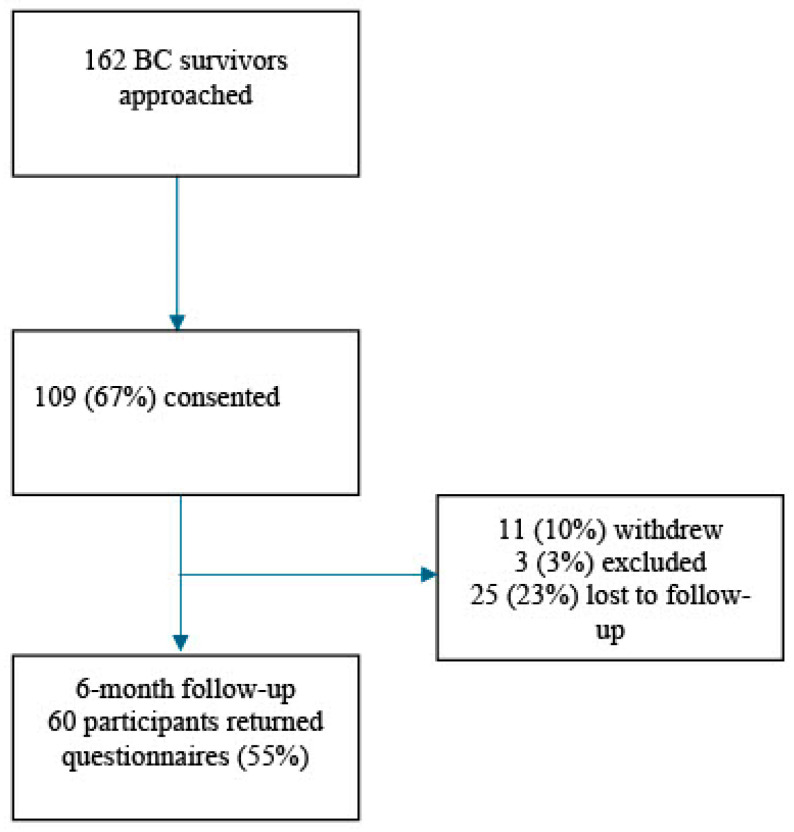
Consort table.

A total of 162 patients were approached to participate in the larger study from which these data are drawn, and 109 (67%) consented (see Figure 1). At the time of T2 follow-up, 70 (64%) participants were able to be contacted and 60 (86%) of them returned the study questionnaires. Of the participants who consented to the larger study, 11 (10%) had withdrawn from the study, 3 (3%) had been excluded, and 25 (23%) were lost to follow-up. No significant differences were found between the participants who returned questionnaires and those who did not in terms of their baseline pain (assessed immediately post-surgery), education, or employment status, or whether or not they underwent axillary node dissection. Significant differences were found between the two groups in terms of age (t = 1.020, *p* = 0.017), ethnicity (χ^2^_(1)_ = 4.169, *p* = 0.041), and marital status (χ^2^_(1)_ = 6.292, *p* = 0.012), such that the participants who returned questionnaires were older (59.8 ± 9.94 vs. 57.5 ± 12.83 years old), more likely to be White (85.0% vs. 77.9%), and more likely to be single (48.3% vs. 38.2%) than those who did not.

#### 3.1.2. Cancer Pain

BPI Pain Intensity (Worst, Least, or Average pain) and Interference scores were not normally distributed (*p* < 0.05). The majority of participants (*n* = 40; 67%) reported some pain in the past 24 h (BPI Worst Pain >0). Of the participants who reported pain, 19 (48%) reported mild pain (BPI Worst Pain 1–3/10), 15 (38%) reported moderate pain (BPI Worst Pain 4–6/10), and 6 (15%) reported severe pain (BPI Worst Pain ≥7/10 [12]).

#### 3.1.3. Pain-Self Management Behaviors

The majority of the participants (*n* = 55; 92%) reported engaging in at least one PSCBQ-PSMB. In addition, during the brief phone interview that the participants completed before receiving their questionnaire package in the mail, 19 (32%) participants reported taking over-the-counter pain medication and 5 (8%) reported taking prescribed pain medication. Participants who reported no pain and who did not report engaging in ≥1 PSCBQ-PSMB (*n* = 5; 8%) were excluded from further analysis described below.

Descriptive statistics of the PSCBQ are presented in Table 2. The total number of PSCBQ-PSMBs and Mean Effectiveness were normally distributed (*p* > 0.05) and Maximum Effectiveness was not (*p* < 0.001). The mean Total Number of PSCBQ-PSMBs was 6.96 ± 3.50 (median = 6.50) out of a possible 25, and the overall Mean Effectiveness was 4.83 ± 2.42 out of 10 (median = 5.00). The mean Maximum Effectiveness had a median of 8.00 and 60.7% (*n* = 34) of participants who reported engaging in at ≥1 PSMB rated at least one PSCBQ-PSMB item as ≥7/10 in terms of effectiveness. The most commonly endorsed PSCBQ-PSMBs were going for a walk (76%), distraction strategies such as reading (76%), listening to music or the radio (76%), and watching TV (75%), and exercise (64%). Most participants (*n* = 40; 67%) who reported engaging in ≥1 PSMB also had Worst Pain scores ≥1. Fifteen participants (25%) who reported engaging in ≥1 PSMB reported a Worst Pain score of zero (indicating no pain in the past 24 h).

#### 3.1.4. Examining the Relationship Between PSCBQ and Pain Intensity and Interference

Table 3 displays the correlations between the BPI Pain Intensity and Interference and the PSCBQ Total Number of PSMB, and Mean and Maximum Effectiveness scores. No significant correlations were found between pain intensity and any of the three PSCBQ scores. However, a significant positive but low correlation was found between Pain Interference and the Total Number of PSMBs (r_s_ = 0.299, *p* = 0.028).

The participants who reported engaging in exercise (PSCBQ item 3) reported significantly lower BPI Worst Pain scores (*M* = 2.26, *SD* = 2.43) than the participants who did not engage in exercise (*M* = 3.89, *SD* = 2.88), *p* < 0.05. The participants who reported using a heating pad or hot water bottle (PSCBQ item 11) reported significantly higher BPI Worst Pain scores (*M* = 5.09, *SD* = 2.88) than the participants who did not (*M* = 2.25, *SD* = 2.34), *p* < 0.01. The participants who reported using an ice pack (PSCBQ item 12) reported significantly higher BPI Worst Pain scores (*M* = 6.60, *SD* = 2.41) than the participants who did not (*M* = 2.45, *SD* = 2.42), *p* < 0.01. No other differences in BPI Worst Pain were found between the participants who engaged/did not engage in any other PSMB as per the PSCBQ.

### 3.2. Qualitative Outcomes

**Figure 2:**
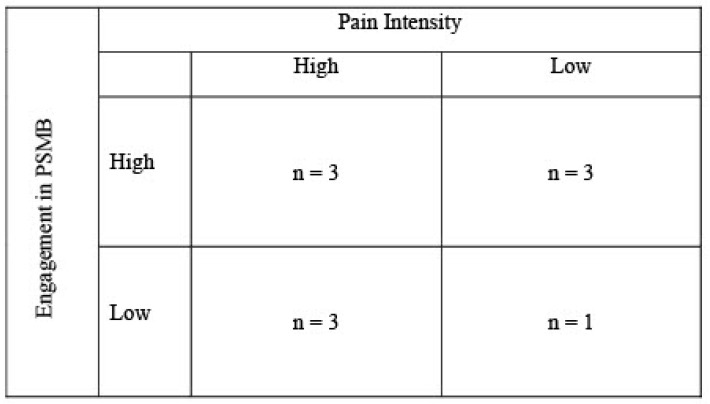
Distribution of qualitative participants based on purposive sampling. Note: pain intensity was measured using BPI Worst Pain and engagement in PSMBs was measured using the PSCBQ subscale ‘Total Number of PSMB’.

Purposive sampling resulted in interviews with three participants who had high pain (≥4/10 BPI Worst Pain) and were high engagers (>above sample mean for total number of PSCBQ-PSMBs); three who had high pain and were low engagers; three who had low pain and were high engagers; and one with low pain who was a low engager (see Figure 2). No new themes were identified among the total sample of women who participated in the qualitative interviews after the sixth interview, and four additional interviews were conducted thereafter to ensure adequate representation across purposive sampling categories [60].

#### 3.2.1. Pain Self-Management Behaviors

Participants reported engaging in PSMBs that fell under eight different categories, listed in Table 4 from the most to the least commonly reported. These categories were created by using the existing literature on common PSMBs [31,57,58,61] as a guide to collapse similar items on the PSCBQ. Most categories overlapped with PSCBQ items, with the exception of three new categories that emerged from the interviews, including avoidance, seeing pain specialists (e.g., attending lymphedema clinics), and the use of topical agents. The interview findings supported the quantitative results while providing context to quantitative responses, and also introduced new PSMBs that were not captured by the PSCBQ. For example, walking and general exercise, which were two of the most commonly endorsed items on the PSCBQ, were also the most commonly discussed during interviews under the category of ‘physical activity’. The PSCBQ’s exercise item includes the examples of jogging and swimming (i.e., “Did exercises (jogging, swimming etc.)”), which may be interpreted as leisure activities that may or may not require a provider with specific expertise. However, during interviews, participants described additional physical activities not captured by the PSCBQ, such as rehabilitative exercises including arm stretching and strengthening exercises and breast self-massage for lymphedema, which likely require the guidance of a provider with specific expertise (e.g., a physiotherapist). Further, although the PSCBQ limits distraction activities to watching TV and listening to music, during interviews, participants also described socializing with friends under the category of ‘distraction’. While only 3.6% (*n* = 2/55) endorsed taking tranquilizers to help with pain on the PSCBQ, forty percent (*n* = 4/10) of participants who completed semi-structured interviews described taking pain medication, suggesting that the item wording on the PSCBQ (i.e., “If you did any of the following things in the past week to help your pain, mark YES”: ‘Took tranquilizers’) may underrepresent pharmacologic pain management.

#### 3.2.2. Goals for Pain Relief and Referral Sources

Table 4 also lists goals for pain relief and sources of PSMB referral. The participants’ goals for pain relief mainly involved minimizing pain interference. For example, participants discussed taking over-the-counter pain medication only when their pain was severe enough to interfere with activities such as sleep or work. Participants also discussed the goal of minimizing pain-related disability or interference and described engaging in rehabilitative exercises such as arm stretching exercises recommended by their doctor with the goal of releasing muscle tension. Further, participants described goals related to relaxation and distraction strategies such as shifting their focus away from pain, which indirectly impacted the pain intensity and interference. Most PSMBs were self- or peer-referred, but participants also described receiving information about PSMBs from HCPs such as their family doctor or radiation oncologist.

## 4. Discussion

To our knowledge, this is the first in-depth exploration of the self-reported PSMBs of survivors of BC using mixed methods. On average, the participants reported engaging in seven PSCBQ-PSMBs. The commonly endorsed PBSCQ items were rated as moderately effective for pain relief and the majority of participants (60%) found at least one PSCBQ item to be very effective for pain relief (i.e., ≥7/10). The most commonly reported PSCBQ items were walking, distraction strategies such as listening to music, and general exercise. Pain intensity was not associated with engagement in the PSCBQ-PSMBs; however, pain interference was positively associated with the total number of PSMBs on the PSCBQ. In addition, passive PSCBQ-PSMBs like using a heating pad or ice pack were associated with higher pain, and active PSCBQ-PSMBs like exercise were associated with lower pain.

In some instances, qualitative data supported the quantitative findings and helped expand our understanding of PSMBs by providing context to the items on the PSCBQ. In other instances, qualitative data also revealed PSMBs that were not captured by the PSCBQ and indicted that there was a poor fit between some participant-reported PSMBs and items on the PSCBQ (e.g., taking pain medication). During the semi-structured interviews, participants commonly reported engaging in physical activity and described it as moderately effective for pain relief. In addition to walking and general exercise, as assessed by the PSCBQ, participants further described engaging in arm stretching and strengthening exercises, breast self-massage, cardio, and yoga as PSMBs. This is consistent with Berkowitz et al. [62], who found exercise to be helpful for survivors of BC experiencing cancer treatment-related side effects. However, physical and exercise-related self-management strategies for pain have received little attention in the survivorship literature [58]. In a review exploring self-management in patients with cancer, Howell et al. [57] identified only three studies that listed physical or exercise-related components, but their effectiveness for pain relief was equivocal [63,64,65]. Our qualitative findings elucidate additional PSMBs that are not included in the PSCBQ and indicate that physical exercises may include activities unique to survivors of BC, such as specific stretching regimens, which usually require intervention from a provider with expertise in rehabilitative exercises after cancer treatment. Future efforts to revise the PSCBQ may consider distinguishing leisure exercise activities from structured exercises derived from a rehabilitation program.

During interviews, participants also commonly discussed engaging in relaxation and distraction to manage their pain. Distraction involves activities that divert attention away from pain and improve mood such as listening to music, watching TV, and reading [66]. Distraction-related activities were the second most commonly reported PSMBs via the PSCBQ and over two-thirds of participants reported engaging in distraction-related PSMBs on the PSCBQ. During interviews, participants described distraction activities that are not included in the PSCBQ such as socializing with friends. Distraction strategies were rated as moderately effective via the PSCBQ. However, the existing literature reviews on self-management interventions for cancer pain do not report on the use or effectiveness of distraction strategies (e.g., [31,33,67]). In post-operative patients with BC, distraction strategies such as listening to music have been found to effectively improve pain and this is thought to occur through reduced attention to pain and increased relaxation [68]. Given the high prevalence of engagement in distraction and the moderate effectiveness of distraction for pain relief reported by participants in the present study, further research on distraction as a PSMB in disease-free survivors of BC post-treatment is warranted.

Relaxation strategies were also commonly reported among participants in both the quantitative and qualitative components of this study. Many participants reported engaging in relaxation exercises or meditating via the PSCBQ. Similarly, during interviews, participants described engaging in deep breathing and positive thinking activities. Participants discussed how these types of PSMBs helped to shift their focus to positive or neutral thoughts and facilitated comfort with an ultimate goal of reducing pain-related distress. Although the use of relaxation strategies as PSMBs among cancer survivors has received very little attention [58], participants in the present study perceived them to be moderately effective. Within the affective PSMB category, participants also described using meditation activities as a way to shift their focus to positive or neutral thoughts. However, it was unclear if participants engaged in meditation to focus on the present moment (as in the case of mindfulness meditation) or if they engaged in meditation as a distraction strategy through the use of guided imagery, for example. With respect to the evidence base for mindfulness meditation, a recent systematic review found no evidence to support its use for cancer pain [69], perhaps in part due to methodological weaknesses and inconsistent operationalizations of mindfulness meditation across studies [70]. Future research is needed to understand how survivors of BC are selecting and using meditation as a PSMB to guide the development of effective, provider-supported multimodal pain self-management strategies.

In interviews, participants expanded on and described several additional PSMBs to those listed on the PSCBQ. For example, participants discussed visiting medical professionals such as physiatrists and lymphedema specialists. Participants also commonly described using over-the-counter pain medication such as ibuprofen and acetaminophen, as well as topical agents such as vitamin E cream to manage their pain. Further, participants described additional assistive devices to those listed on the PSCBQ including sit-stand desks and cervical collars. Avoidance emerged as a new category during interviews and one participant described anxiety related to movement of her arm. In addition, there was also a discrepancy between the use of pain medication reported via the PSCBQ and that reported in interviews. Less than 4% of participants reported using ‘tranquilizers’ on the PSCBQ but almost half of those who participated in the interview discussed using over-the-counter, and in one instance, prescribed pain medication. Although we did not collect data on how participants interpreted items on the PSCBQ, it is possible that they interpreted the word ‘tranquilizer’ differently than pain medication. Consequently, the use of pain medication as a self-management behavior may be underreported in studies that derive this information solely from responses to this item on the PSCBQ.

Interestingly, pain intensity was not correlated with engagement in the PSCBQ-PSMB. In addition, and in contrast with what might be expected, pain interference was positively associated with Total PSCBQ-PSMB. These unexpected findings are inconsistent with the literature describing the relationships between intensity and interference of other cancer-related symptoms and engagement in self-management behaviors for those symptoms. For example, physical activity and relaxation have been associated with reduced sleep disturbance and fatigue-related interference [71,72]. In the case of cancer-related pain, it may be that people who have higher pain interference are more likely to try multiple strategies to self-manage their pain, with varying levels of success.

Several possibilities can be considered in interpreting the lack of relationship between the PSCBQ-PSMBs and pain intensity. It may be possible that the PSCBQ does not capture the key PSMBs in which survivors of BC engage and find most effective, or that survivors lack knowledge about how to effectively perform PSMBs. However, most participants reported at least one PBSCQ item to be very effective for pain relief, suggesting that there may be other explanations for the lack of association. When asked about goals for pain relief during interviews, most participants discussed the objective of minimizing their pain’s impact or interference, with some participants explicitly describing goals such as “release muscle tension”, “improve range of motion”, and “shift focus to positive or neutral thoughts”. Therefore, an alternate possibility is that the desired outcome of engagement in PSMBs is not lower pain, but rather, lower interference from pain. If this is true, it would provide additional support for our interpretation of the positive relationship between pain interference and Total PSCBQ-PSMB. Related, studies of cognitive and behavioral therapies for chronic non-cancer pain, such as acceptance-based treatments, have consistently shown long-term reductions in pain-related interference in the absence of a prolonged reduction in pain intensity [73,74]. While existing studies of PSMBs for cancer are generally concerned with decreasing pain intensity [33], the most clinically important outcome may instead be pain interference. Future research testing the effectiveness of PSMBs for people with and after cancer should incorporate pain interference as a clinically meaningful outcome measure.

In addition to expanding our understanding of the relationship between pain and PSMBs as reported on the PSCBQ, the qualitative interviews also broadened our understanding of the referral sources of many of these PSMBs. Most PSMBs were self-directed or suggested by friends. Although one study has suggested that HCPs are patients’ preferred CAM referral source [39], these findings are not consistent with several other studies of CAM decision-making regarding cancer symptoms or chronic pain [62,75,76], which found that the preferred referral sources are not HCPs, but rather friends, family, and other sources with unclear healthcare knowledge and expertise. Importantly, the effectiveness of several of the reported strategies is uncertain and the quality of evidence supporting some strategies is low (e.g., acupuncture, chiropractic care, mindfulness meditation) [69,77,78,79].

Torresan et al. [80] have suggested that self-management promotes shared decision-making with HCPs to align treatment with patient preferences. Our data indicate that this may not be true where referral strategies were self-directed or suggested by friends. In a large study of over 2300 survivors of BC, 31.5% reported feeling unsupported by their healthcare team regarding the management of cancer treatment side-effects [62]. Similarly, in women with BC, Bender et al. [81] found that participants reported unmet needs related to information on what to expect from cancer pain, how to describe it to their HCPs, options for pain control, and help managing it. These findings were recently confirmed in a meta-ethnography of qualitative studies describing patients’ cancer pain self-management needs [82], where significant gaps were noted in the education and information available to help patients make pain management decisions, especially in cases of multimodal treatment strategies. Taken together, these data may suggest that preference for family/friends as referral source may, in some cases, reflect the fact that these are the only or default referral source of PSMBs. Critically, they highlight a serious gap in care and an important area of focus for future research to determine survivors’ decisional needs and HCPs’ needs to support survivors in making value- and evidence-based pain management decisions.

While this is the first study to describe the PSMBs of survivors of BC using a mixed-methods approach, the findings need to be interpreted in the context of its limitations. First, the PSCBQ has yet to undergo extensive psychometric testing and, thus, this study was limited by the use of a non-validated outcome measure. However, the PBSCQ is the only available measure of PSMBs developed for use in people with cancer-related pain, and thus provides important information about people’s attempts to self-manage their pain and the effectiveness of attempted strategies. Second, we did not collect data regarding the frequency or duration of PSMBs. This information may be particularly relevant for some PSMBs such as exercise, as well as the maintenance of the attempted strategies, which may influence the scores derived from the PSCBQ. Thus, future research should consider these important factors. Third, this study had a relatively small sample size and used bivariate analysis, which did not allow for inferences about the direction of significant correlations. Further, our response rate was relatively low and, therefore, this study may be subject to participation bias. Fourth, the participants were recruited from Canada’s largest cancer center, which is relatively well-resourced in terms of rehabilitation and exercise programs [83] compared to some other community centers where such programs are not available, and our sample was primarily White and highly educated. Accordingly, caution should be taken in applying the findings from the present study to other survivors of BC.

Despite these limitations, this study is heuristic and expands our understanding of PSMBs in survivors of BC. The inclusion of qualitative data allowed us to gain a deep, contextual understanding of the use of PSMBs by survivors of BC [84]. Specifically, semi-structured interview data confirmed many of the strategies included on the PSCBQ, and importantly, expanded our understanding of the repertoire of strategies in which survivors of BC engage to manage their pain, which may contribute to future efforts to define and better support PSMBs. In addition, they highlighted a potentially important interpretation issue with the item querying tranquilizer use as a pain self-management strategy. These data could guide future efforts to refine the PSCBQ and other PSMB measurement methods, as well as aid sample size calculations for future research on pain self-management behaviors using the PSCBQ.

Future research should build on these findings to better define pain self-management behaviors, to refine their measurement, and inform pain self-management interventions for cancer survivors. This is critical, as current PSMB interventions lack consideration of commonly endorsed PSMBs such as physical activity [33], which may be an effective way to decrease pain in some cancer survivors [63]. The emergence of avoidance as a PSMB provides important information for the development of provider-supported pain self-management strategies and suggests that special attention should be paid to intervention aspects that address fear of movement, which potentially leads to disuse, deconditioning, and greater pain and disability [85,86]. From a measurement perspective, guidelines for the management of pain in the post-treatment survivorship period privilege some PSMBs over opioid use [3,87,88]; thus, future efforts to validate the PSCBQ would provide an important tool to measure the range of behaviors and their effectiveness in self-managing pain. Related, the most commonly used metrics of the quality of pain management, like the pain management index [77,78], completely ignore PSMBs. However, this study and others (e.g., [89,90,91]) suggest that people commonly engage in PSMBs across the cancer trajectory, either in conjunction with analgesic treatment or possibly in lieu of it, where access to prescribers is limited. Thus, it is possible that participation in PSMBs impacts scores on these commonly used metrics. Future research should also consider how to integrate PSMBs in measures of the quality of pain management. Lastly, few existing PSMB interventions are based on a theoretical framework [33] and the SMT [28,29] may be a promising framework to consider.

## 5. Conclusions

Some PSMBs were self-guided, while others required intervention from a HCP or other person (e.g., physiotherapist, acupuncturist) or access to extremely limited resources (e.g., cancer exercise program, physiatrist). Thus, although the full range of barriers and facilitators to the uptake and maintenance of PSMBs has yet to be determined, they are likely present at the individual, HCP, and system levels. Importantly, the evidence base of several strategies does not support their use and, in other cases, the evidence is unclear; therefore, much further research is needed to determine the effectiveness of many PSMBs prior to their widespread clinical implementation [92].

## Figures and Tables

**Table 1:** Participant demographic, clinical, and pain characteristics.

		*n* (%), Range
Age (Mean ± SD)		60.58 ± 9.88
Ethnicity	White	51 (85.0)
	Asian	7 (11.7)
	Other	2 (3.3)
Education	High School	9 (15.0)
	College/Undergraduate Degree	37 (61.7)
	Graduate/Professional Degree	14 (23.3)
Marital Status	Married/Partnered	29 (48.3)
	Single	31 (51.7)
Employment Status	Employed Full or Part-Time	25 (41.7)
Living Arrangement	Alone	21 (35.0)
	With Partner	18 (30.0)
	With Partner and Children	11 (18.3)
	Other	10 (16.7)
Adjuvant Cancer Treatment	Radiation	29 (48.3)
	Chemotherapy	4 (6.7)
	Both	16 (26.7)
Hormone Therapy	Yes	40 (66.7)
Type of Primary Surgery	Lumpectomy	42 (70.0)
	Mastectomy	17 (28.3)
Axillary Node Dissection	Both	1 (1.7)
	Yes	11 (18.3)
Pain Medication	Non-opioid ^a^	19 (31.7)
	Opioid	5 (8.3)
Participants with BPI Worst Pain >0 in Past 24 Hours		40 (66.7)
Participants Engaging in ≥1 PSMB		55 (91.7)
Participants w/BPI Worst Pain >0	Worst Pain	3.93 ± 2.36, 1–10
	Least Pain	2.00 ± 2.22, 0–10
	Average Pain	3.00 ± 2.25, 0–10
	Pain Interference	2.09 ± 2.11, 0–8

^a^ Acetaminophen, non-steroidal anti-inflammatory or acetylsalicylic acid. Notes: BPI, Brief Pain Inventory; PSMB, pain self-management behaviors; pain in past 24 h measured via Worst Pain ≥1/10 on the BPI; engagement in PSMB measured via ≥1/25 on the Pain Self-Care Behaviors Questionnaire.

**Table 2:** Descriptive statistics of the Pain Self-Care Behaviors Questionnaire (PSCBQ).

PSCBQ Item	*n* (%) of Participants Who Endorsed Item	Mean Effectiveness (SD)	Median Effectiveness	*n* (%) of Participants Who Rated Item as Most Effective
Went for a walk	42 (76.4)	5.10 ± 3.15	6.00	12 (21.8)
Read a book, newspaper or magazine	42 (76.4)	4.53 ± 3.01	4.50	8 (14.5)
Listened to radio, music	42 (76.4)	4.49 ± 2.94	5.00	8 (14.5)
Watched TV	41 (74.5)	4.79 ± 2.98	5.00	10 (18.2)
Did exercises (jogging, swimming etc.)	35 (63.6)	4.89 ± 2.65	5.00	10 (18.2)
Did relaxation exercises, meditated	26 (47.3)	4.88 ± 2.68	5.50	6 (10.9)
Took a nap	25 (45.5)	5.08 ± 2.64	5.50	7 (12.7)
Reduced my level of activity	22 (40.0)	4.73 ± 2.68	5.00	6 (10.9)
Took a hot bath	18 (32.7)	5.39 ± 2.48	5.00	7 (12.7)
Drank beer, wine, or other alcohol	17 (30.9)	3.57 ± 3.16	3.00	1 (1.8)
Had a massage	14 (25.5)	7.54 ± 1.45	8.00	7 (12.7)
Used a heating pad or hot water bottle	11 (20.0)	4.90 ± 2.60	4.50	3 (5.5)
Reduced hours at work	10 (18.2)	5.11 ± 3.06	5.00	4 (7.2)
Asked for help	10 (18.2)	4.60 ± 2.76	5.00	4 (7.2)
Used assistive devices	6 (10.9)	5.83 ± 1.94	5.50	3 (5.5)
Used an ice pack	5 (9.1)	3.80 ± 2.17	3.00	0 (0)
Went to a chiropractor	4 (7.3)	7.00 ± 1.83	7.00	3 (5.5)
Used a TENS	3 (5.5)	7.50 ± 0.71	7.50	2 (3.6)
Went for acupuncture treatment	3 (5.5)	5.33 ± 1.56	6.00	1 (1.8)
Went for counseling	3 (5.5)	4.67 ± 4.16	6.00	0 (0)
Took tranquilizers	2 (3.6)	6.50 ± 2.12	6.50	1 (1.8)
Ultrasonic stimulation treatment	1 (1.8)	1.00	-	0 (0)
Used magnets	1 (1.8)	1.00	-	0 (0)
Had a trigger point injection	0 (0)	-	-	-
Did hypnosis	0 (0)	-	-	-

Note: Mean Effectiveness was calculated using only the scores of participants who endorsed engaging in that item.

**Table 3:** Correlation matrix.

	Mean ± SD	1	2	3	4	5	6
1. PSCBQ Total Number of PSMB	6.96 ± 3.50						
2. PSCBQ Mean Effectiveness	4.83 ± 2.42	−0.141					
3. PSCBQ Max Effectiveness	6.71 ± 2.59	−0.011	0.741 ^b^				
4. BPI Worst Pain	2.85 ± 2.67	0.071	−0.213	−0.187			
5. BPI Least Pain	1.47 ± 2.08	0.022	−0.156	−0.101	0.749 ^b^		
6. BPI Average Pain	2.20 ± 2.33	0.060	−0.180	−0.144	0.949 ^b^	0.847 ^b^	
7. BPI Pain Interference	1.60 ± 2.01	0.299 ^a^	−0.149	−0.156	0.751 ^b^	0.599 ^b^	0.760 ^b^

Note: PSCBQ (Pain Self-Care Behaviors Questionnaire; BPI (Brief Pain Inventory). ^a^ Two-tailed (*p* < 0.05); ᵇ Two-tailed (*p* < 0.01).

**Table 4:** Pain self-management behaviors (PSMBs) reported during interviews.

Category	Example(s)	Referral Source	Primary Goal(s)	Demonstrative Quote(s)
Physical Activity	Arm stretching, strengthening exercises Breast Self-Massage (for lymphedema and tightness) General strengthening exercises or weight training Cancer-related exercise programs	Radiation Oncologist and other unspecified HCPs	Release muscle Tension Improve circulation Distraction	“*And I’ll, you know, I might be in the kitchen and sort of walk my arm up the cupboard and down. Just to sort of release and stretch...*” -Participant 6 “*I feel better if I’m jogging. I think it’s because of the circulation... Because after jogging I feel like my body feels better.*” -Participant 5 “*The strategy, believe it or not, it’s exercise... it’s [getting] up and walking... Keep yourself moving. And I find the exercise, or just the moving, also helps me not concentrate on the pain as much. So that also helps me.*” -Participant 3
	Yoga Walking Jogging	Self		
Relaxation	Meditation Heating pad Deep breathing Laugh therapy Positive thinking	Self	Shift focus to positive or neutral thoughts Comfort	“*It really helped me lessen the pain... I focused on just a lot of positive things*.” -Participant 4 “*I mean they’re not getting rid of it but, particularly the heating pad when I’m sitting—I don’t use it in bed—but when I’m sitting in a chair reading, I put it at the base of my spine and, yah, it makes me feel comfortable.*” -Participant 7
Pain Medication	Hydromorphone	Family Doctor	Minimize daily pain interference	“*I’ll only take the Advil after the pain has been severe enough. And usually never at home... Usually if I have to take the Advil it’s while I’m at the office. And that only happens when the pain is such that it actually interferes with me being able to do my job. So if I’m sitting and the pain is just too much that I can’t sit for too long or I can’t concentrate on my work because of the pain... So I’m trying to minimize, I’m not letting my injury or whatever you want to call this, get any worse.*”-Participant 3
	Acetaminophen Ibuprofen	Self		
Medical Professionals	Physiotherapist Lymphedema clinic Physiatrist Massage therapist	Social Network	Improve range of motion; arm strength Decrease swelling (due to lymphedema)	“*Doing the exercises the physiotherapist gave me, it was very helpful. Just in terms of slowly increasing the range of motion and the strength in that arm.*” -Participant 10
Distraction	Socializing with friends General activity	Self	Shift focus away from pain	“*But I do go out with pain and I do have a good time because it takes my mind off myself. So, you know, I do like to socialize. People come here or I go somewhere. And it takes my mind off of everything for a while... and that relieves it for a bit, whatever time I’m with somebody, you know, a couple of hours or whatever.*” -Participant 7
Topical agents	Vitamin E	Radiation Oncologist	Help facilitate other PSMB (i.e., massage)	“*So after I spoke to my oncologist, he told me that maybe I could try the vitamin E oil, so I bought the vitamin E oil, and after I put the vitamin E cream on top to massage, so it’s easy to spread. Then, I found that after the week, all the hard tissue [was] gone*.” -Participant 5
	A535 cream	Family Doctor		
Avoidance	Not using arm on side of surgery	Self	Limit activity to minimize chances of experiencing pain	“*I’m not eager to use my arm. I try to minimize that, anticipating pain... I just avoid*.” -Participant 2
Assistive Devices & other Non-Invasive Adjuvant treatments	Cervical collar Sit-stand desk	Family Doctor	Minimize pain interference	-
	Compression stockings Acupuncture Transcutaneous electric nerve stimulation Wearing a bra	Self		

Note: HCPs (healthcare providers).

## Data Availability

The data sets generated and/or analyzed during the current study are not publicly available due to ethical restrictions but are available from the corresponding author on reasonable request.

## Abbreviations

The following abbreviations are used in the manuscript:

BC	breast cancer
BPI	Brief Pain Inventory
CAM	complementary and alternative medicine
CTP	chronic treatment-related pain
PSCBQ	Pain Self-Care Behaviors Questionnaire
PSMB	pain self-management behaviors
HCP	healthcare provider
SMT	Symptom Management Theory
SOMC	Short Orientation Memory Concentration test

## Appendix A

### Appendix A.1. Interview Guide

General questions pertaining to experiences with pain:

1. Can you tell me about your experience with pain

 Probing points:

 (a) When did your pain start?

 (b) Where do you have pain?

 (c) What does it feel like?

 (d) How much does it hurt?

2. Do you feel that the pain you experience affects your day-to-day life and if so, in what ways?

 (a) How do you usually deal with these disruptions?

Pain Self-Management Behaviors:

3. Do you use any strategies to help manage your pain? If so, what do these include?

 (a) How did you come to know about these strategies?

 (b) Are these strategies helping you relieve your pain?

 (c) What are your goals for pain relief?

 (d) Which of these pain management strategies (if any) did you find particularly helpful in managing your pain? Which did you not find helpful?

  i. Why? (i.e. Did they relieve pain? Decrease stress? Improve daily functioning?)

 (e) At what point did you decide to take/engage in (analgesics/X pain management strategy)?

 (f) What do you believe about (analgesics/X pain management strategy)and how they/it can help you to feel better?

 (g) How do (analgesics/X pain management strategy)make you feel?

If participant takes pain medication...

 (a) Do you take pain medication as directed? If yes, why? If no, why not?

 (b) Were you given directions on how to take the medication?

Barriers and Facilitators

4. Have you experienced any challenges (barriers) managing your pain?

 Probing for barriers re: strategies:

 (a) Have you experienced any difficulty engaging in the strategies you discussed a moment ago? If so, what do these difficulties include?

  i. Were there any stress or emotional factors that made using these strategies easier or harder for you?

  ii. Were there any physical limitations or illness factors that made using these strategies harder?

 Probing for facilitators re: strategies:

 (a) What are some of the things that made these strategies easy to use regularly?

  i. Are there social supports or resources in your life that made engaging in pain relieving activities easier or more difficult?

Access to Pain Self-Management Interventions

5. Would further information on pain relief strategies be of interest to you?

If YES.

 (a) What types of pain relief strategies would you be interested in trying?

 (b) What would help to facilitate your participation in them

  Probing point:

  Is there a particular delivery method you would prefer (in person, in groups, online, alone, via pamphlets and other printed materials)?

6. If you could tell your health care team how better to treat your pain, what would you tell them?
